# Release Profile and Antibacterial Activity of *Thymus sibthorpii* Essential Oil-Incorporated, Optimally Stabilized Type I Collagen Hydrogels

**DOI:** 10.3390/bioengineering12010089

**Published:** 2025-01-19

**Authors:** Caglar Ersanli, Ioannis Skoufos, Konstantina Fotou, Athina Tzora, Yves Bayon, Despoina Mari, Eleftheria Sarafi, Konstantina Nikolaou, Dimitrios I. Zeugolis

**Affiliations:** 1 Laboratory of Animal Science, Nutrition and Biotechnology, School of Agriculture, University of Ioannina, 47100 Arta, Greece; c.ersanli@uoi.gr (C.E.); jskoufos@uoi.gr (I.S.); 2 Laboratory of Animal Health, Food Hygiene and Quality, School of Agriculture, University of Ioannina, 47100 Arta, Greece; kfotou@uoi.gr (K.F.); knikolaou@uoi.gr (K.N.); 3 Regenerative, Modular & Developmental Engineering Laboratory (REMODEL), Charles Institute of Dermatology, Conway Institute of Biomolecular and Biomedical Research and School of Mechanical and Materials Engineering, University College Dublin (UCD), D04 V1W8 Dublin, Ireland; dimitrios.zevgolis@ucd.ie; 4 Medtronic—Sofradim Production, 116 Avenue du Formans—BP132, F-01600 Trevoux, France; yves.bayon@medtronic.com; 5 Department of Biological Applications & Technology, School of Health Sciences, University of Ioannina, 45110 Ioannina, Greece; d.mari@uoi.gr (D.M.); e.sarafi@uoi.gr (E.S.); 6 Biomedical Research Institute, Foundation for Research and Technology-Hellas, 45110 Ioannina, Greece

**Keywords:** antibacterial hydrogel, essential oil, *Thymus sibthorpii*, collagen, antimicrobial resistance, tissue engineering

## Abstract

Antimicrobial resistance is one of the drastically increasing major global health threats due to the misuse and overuse of antibiotics as traditional antimicrobial agents, which render urgent the need for alternative and safer antimicrobial agents, such as essential oils (EOs). Although the strong antimicrobial activity of various EOs has already been studied and revealed, their characteristic high sensitivity and volatility drives the need towards a more efficient drug administration method via a biomaterial system. Herein, the potential of *Thymus sibthorpii* EO incorporated in functionalized antibacterial collagen hydrogels was investigated. At first, the optimally stabilized type I collagen hydrogels via six different multi-arm poly (ethylene glycol) succinimidyl glutarate (starPEG) crosslinkers were determined by assessing the free amine content and the resistance to enzymatic degradation. Subsequently, 0.5, 1, and 2% *v*/*v* of EO were incorporated into optimized collagen hydrogels, and the release profile, as well as release kinetics, were studied. Finally, biomaterial cytocompatibility tests were performed. *Thymus sibthorpii* EO was released from the hydrogel matrix via Fickian diffusion and showed sustained release and 0.5% *v*/*v* EO-loaded hydrogels showed adequate antibacterial activity against *Staphylococcus aureus* and did not show any statistically significant difference compared to penicillin (*p* < 0.05). Moreover, none of the fabricated composite antibacterial scaffolds displayed any cytotoxicity on NIH-3T3 fibroblasts. In conclusion, this work presents an innovative antibacterial biomaterial system for tissue engineering applications, which could serve as a promising alternative to antibiotics, contributing to coping with the issue of antimicrobial resistance.

## 1. Introduction

Antibiotics have generally been the first-line treatment for microbial infections due to their low toxicity and great bactericidal features [1,2,3,4]. Despite their superior biological efficacies, the overuse and misuse of antibiotics have contributed to the promotion of microbes’ antimicrobial resistance, which has emerged as one of the major global health concerns according to the World Health Organization (WHO) [5]. In 2019, it was reported that, annually, 33,000 and 35,000 deaths were caused due to antibiotic-resistant infections in the European Union countries [6] and the United States [7], respectively. On the other hand, high concentrations of antibiotics may be required to treat infections caused by biofilm-forming bacteria, since lower concentrations of antibiotics have led to the enhancement of drug resistance [8,9,10]. However, high doses of antibiotic utilization may cause several adverse effects to the host, such as toxicity in the non-target area and allergies [11,12,13,14]. In consequence, safer and innovative antimicrobial treatment approaches [15] utilizing natural alternatives, such as essential oils, have garnered significant attention.

Essential oils (EOs) are plants’ secondary metabolites obtained from various parts (e.g., stem, root and flower) [16,17,18]. They are colored, volatile, aromatic liquids [19,20] with a characteristic strong odor, demonstrating complex chemical compositions that offer a broad spectrum of antimicrobial activity [21], making them promising candidates in the battle to combat antimicrobial resistance. *Thymus sibthorpii* EO is derived from the *Thymus sibthorpii* plant, a species of the genus *Thymus* that belongs to the family *Lamiaceae,* which mainly inhabits Southeastern Europe. *Thymus sibthorpii* EO has a complex chemical composition that mainly consisted of carvacrol, thymol, and p-cymene [22]. Although, in the literature, its superior antimicrobial, anti-biofilm [22], and antioxidant [23] activities have already been described due to its high volatility and sensitivity, it would be more effective within a biomaterial system [24]. In this context, it is crucial to develop a cytocompatible scaffold as a carrier of the EO, which should also present controlled EO release features. To our best knowledge, *Thymus sibthorpii* EO has not been introduced yet within medical devices in the field of tissue engineering applications, according to the literature.

Collagen, a fibrous, non-soluble protein, is one of the most prominent polymers for the development of antimicrobial biomaterials, attributable to its superior biocompatibility, excellent biodegradability, hydrophilic nature, reduced cytotoxicity, and high cell attachment affinity [25,26,27,28,29,30]. Nevertheless, the fabricated forms of collagen need to be functionalized via in situ crosslinking due to a lack of stability [31,32]. Since physical and biological crosslinking mechanisms have generally resulted in low crosslinking efficacy, carboxyl and amine terminal crosslinking strategies are favored in research, as arises in the literature [33]. Among the above, since carbodiimide and glutaraldehyde often cause cytotoxicity [34,35], alternative crosslinkers such as multi-arm, star-shaped poly(ethylene glycol) succinimidyl glutarate (starPEG) have emerged as the subject of research [33,36,37,38,39]. For instance, Collin et al. indicated that collagen-based hydrogels crosslinked with four-arm starPEG molecules showed no toxicity for adipose-derived stem cells [39].

In this study, at first, collagen type I hydrogels crosslinked with six different starPEG molecules were developed and optimized. Accordingly, the optimally starPEG-crosslinked collagen hydrogels were loaded with several concentrations of *Thymus sibthorpii* EO, and the release profile and kinetics of the EO were investigated. Finally, the antimicrobial activity of the developed composite hydrogels was assessed against *Staphylococcus aureus* (*S. aureus*) and *Escherichia coli* (*E. coli*), whilst their cytocompatibility was examined on the NIH-3T3 fibroblast cell line.

## 2. Materials and Methods

### 2.1. Materials

Porcine dermis pepsinized collagen type I with a purity over 99% was provided by Medtronic (Trevoux, France). *S. aureus* (ATCC^®^ 29213) and *E. coli* (ATCC^®^ 25922) were purchased from the American Type Culture Collection (Manassas, Virginia, USA). The NIH-3T3 mouse fibroblast cell line (ATCC, CRL-1658) was provided by the Department of Biological Applications and Technology, School of Health Sciences, University of Ioannina (Ioannina, Greece). Four-arm PEG succinimidyl glutarate and pentaerythritol (10 and 20 kDa) eight-arm PEG succinimidyl glutarate and hexaglycerol (10 and 20 kDa) and eight-arm PEG succinimidyl glutarate and tripentaerythritol (10 and 20 kDa) were purchased from JenKem Technology USA (Allen, TX, USA). Phosphate buffered saline (PBS, P4417), sodium hydroxide (NaOH, S8045), sodium bicarbonate (NaHCO_3_, S5761), calcium chloride (CaCl_2_, C1016), Dulbecco’s modified Eagle’s mediumhigh glucose (DMEM, D6429), fetal bovine serum (FBS, F7524), penicillinstreptomycin (P/S, P4333), trypan blue (T8154), and Dulbecco’s phosphate-buffered saline (DPBS, D1408) were obtained from Sigma Aldrich (Athens, Greece). 2,4,6-trinitrobanzene sulfonic acid (TNBSA, 28997), glutaraldehyde (GTA, 119980010), sodium dodecyl sulfate (SDS, S/P530/53), glycine white crystals (BP381), collagenase type II from *Clostridium histolyticum* (Gibco^TM^, 17101-15), tris-base (BP152), a Pierce^TM^ BCA protein assay kit (23227), trypsin-EDTA (0.25%, 25200-056), an alamarBlue^TM^ assay kit (Invitrogen^TM^, DAL1100), and a Quant-iT^TM^ PicoGreen^TM^ dsDNA assay kit (Invitrogen^TM^, P11496) were purchased from Thermo Fisher Scientific (Athens, Greece). Acetic acid (33209) and hydrochloric acid (HCL, 30721) were ordered from Honeywell, Fluka^TM^ (Seelze, Germany). All tissue culture plasticware was purchased from Sarstedt (Nümbrecht, Germany).

### 2.2. Fabrication and Crosslinking of Collagen Type I Hydrogels

Collagen type I hydrogels were prepared at a volume of 300 µL. For this reason, type I collagen was dissolved in 0.05 M acetic acid at a final concentration of 5 mg/mL. The pH of the solution was adjusted between 7.1 and 7.4 using 1 N NaOH and 10× phosphate-buffered saline (PBS). Then, stock crosslinker solution was added to the mixture at the desired final concentration. The final mixture was incubated at 37 °C for 1 h in a humidified atmosphere of 5% CO_2_ for complete gelation. Several types of multi-arm, star-shaped PEG succinimidyl glutarate molecules (Table 1) with different functional groups were used as crosslinking agents at concentrations of 0.5 mM, 1 mM, 2 mM, and 5 mM. Glutaraldehyde (GTA) at a concentration of 0.625% *w*/*v* was used as a positive control [40], whilst non-crosslinked (NCL) hydrogels were determined as a negative control.

### 2.3. Screening of the Crosslinking Efficacy of starPEG Crosslinkers on Collagen Type I Hydrogels

#### 2.3.1. Quantification of the Free Amine Groups

The remaining primary free amines of the collagen type I hydrogels were quantified by using the TNBSA assay, as previously described [41]. The linear standard curve was prepared by using known concentrations of glycine (Figure S1). The fabricated hydrogels were incubated in 0.1 M of sodium bicarbonate at pH 8.5. Then, 0.01% *w*/*v* of TNBSA was added, which was diluted in 0.1 M sodium bicarbonate, and the samples were incubated for 2 h in a 37 °C incubator. Just after that, the reaction was stopped by adding 10% *w*/*v* of SDS and 1 M of HCl. The absorbance of each sample was assessed at 335 nm by a microplate reader (BioTek Synergy HT, BioTek Instruments Inc., Winooski, VT, USA), and the free amine groups were quantified by using the linear standard curve to find the concentration that corresponds to their absorbance.

#### 2.3.2. Enzymatic Degradation Analysis

The resistance of fabricated hydrogels to proteolytic degradation was examined using a collagenase assay, as has been described previously [41], with slight modifications. Briefly, hydrogels were placed into microcentrifuge tubes for each experimental group and each time point (0, 2, 4, 8, and 24 h). Then, 0.1 M Tris-HCl buffer at pH 7.4, and 50 Units/mL degradation buffer prepared from collagenase type II extracted from *Clostridium histolyticum* were added to the samples in equal volumes. All samples were incubated at 37 °C on a horizontal orbital shaking incubator at 150 rpm. At each defined time point, the supernatant was collected and transferred into a new microcentrifuge tube. The amount of dissolved collagen was assessed using the Pierce^TM^ BCA protein assay, as per the manufacturer’s protocol.

### 2.4. Release Profile and Release Kinetics Analysis of Essential Oil

*Thymus sibthorpii* EO was chosen as an antimicrobial agent based on the detailed antimicrobial and anti-biofilm activity assessment of various EOs in our previous study [22]. Following the screening of various starPEG crosslinkers with different concentrations regarding hydrogel stability, EO was loaded into hydrogels crosslinked with 0.5 mM of 4SP, pentaerythritol, 10 kDa, 4SP, pentaerythritol, 20 kDa, and 8SP, hexaglycerol, 20 kDa. Hydrogels were fabricated as described in Section 2.2. In order to incorporate EO into hydrogels, EO was added to the hydrogel preparation solution with a final concentration of 0.5, 1, and 2% *v*/*v*, and the solution was thoroughly mixed using a benchtop vortex. Then, the final mixture containing the added EO was incubated at 37 °C for 1 h in a humidified atmosphere of 5% CO_2_ for complete gelation.

The release profile of the EO was analyzed, as has been described previously [42], with slight modifications. The fabricated EO-loaded hydrogels were soaked into 1 mL of 1× PBS (pH 7.4) at 37 °C using a horizontal shaker incubator. At each defined time point (0, 0.5, 1, 1.5, 2, 2.5, 3, 3.5, 4, 24, and 48 h), 100 µL of sample was removed and replaced by 100 µL fresh 1× PBS. The linear calibration curve was prepared with different concentrations of *Thymus sibthorpii* EO using 70% *v*/*v* ethanol, which was used as a solvent (Figure S2). Then, the absorbance of the supernatant was measured at 365 nm, and the concentration of the released *Thymus sibthorpii* EO was determined by using the standard curve to find where their concentration corresponds. After spectrophotometric evaluation, the cumulative release percentage of the EO was estimated according to Equation (1), where M_t_ is the released amount of EO at time *t*, and *M*_0_ is the initial EO amount.

(1) $$Cumulative\;Release\;\%=\sum_{t:0}^{t}\frac{M_{t}}{M_{0}}\times 100$$

Besides the cumulative release percentages, we studied the release kinetics according to the release profile of *Thymus sibthorpii* EO. Hence, the zero-order, first-order, Higuchi, Korsmeyer–Peppas, and Hixon–Crowell release kinetics models have been applied to post-burst-release data with Equations (2)–(6) that follow [43,44,45,46,47], where *M*_∞_ indicates the amount of EO at the final time of the measurements, *K* is the release constant, and n is the release exponent.

(2) $$\mathrm{Zero}-\mathrm{order}\;\mathrm{model}:\;\frac{M_{t}}{M_{\infty }}=Kt$$

(3) $$\mathrm{First}-\mathrm{order}\;\mathrm{model}:\;\ln\left(1-\frac{M_{t}}{M_{\infty }}\right)=-Kt$$

(4) $$\mathrm{Higuchi}\;\mathrm{model}:\;\frac{M_{t}}{M_{\infty }}=Kt^{1/2}$$

(5) $$\mathrm{Korsmeyer}-\mathrm{Peppas}\;\mathrm{model}:\;\frac{M_{t}}{M_{\infty }}=Kt^{n}$$

(6) $$\mathrm{Hixson}-\mathrm{Crowell}\;\mathrm{model}:\;{M_{0}}^{1/3}-{M_{t}}^{1/3}=Kt$$

### 2.5. Biological Activity of Essential Oil-Loaded Hydrogels

#### 2.5.1. Antimicrobial Activity

The antimicrobial activity of *Thymus sibthorpii* EO-loaded collagen type I hydrogels was assessed by the Kirby–Bauer disc diffusion method against Gram-positive *S. aureus* ATCC 29213 and Gram-negative *E.coli* ATCC 25922 [48]. Penicillin (10 units) and enrofloxacin (5 µg) discs were used as control antimicrobials. On the other hand, the antimicrobial activity of 0.5, 1, and 2% *v*/*v Thymus sibthorpii* EO was studied as a positive control. All solutions required to fabricate hydrogels were exposed to UV light with an intensity of 40 µW/cm^2^ for 15 min in order to be sterilized prior to the fabrication. The fabricated EO-loaded collagen type I hydrogels were sterilized by UV irradiation for 1 h before their antimicrobial activity assessment.

Briefly, *S. aureus* and *E. coli* were cultured overnight in a 37 °C incubator on blood agar and MacConkey agar, respectively. Then, the bacteria inoculum was prepared with a 1 × 10^8^ CFU/mL concentration for each strain separately and spread on the Muller–Hinton agar plates. Afterwards, sterilized EO-loaded collagen type I hydrogels were placed on the Muller–Hinton agar plates. For each experimental group, three replicates were used. Thereafter, Muller–Hinton agar plates with the microbial inoculum and the hydrogels were incubated overnight at 37 °C. The inhibition zone diameters were measured for the quantitative evaluation, whilst images of the plates were taken for the qualitative evaluation.

#### 2.5.2. Cytocompatibility Analysis

Since 0.5% *v*/*v Thymus sibthorpii* EO-loaded collagen type I hydrogels showed no significant difference compared to penicillin, the study was moved forward with 0.5% *v*/*v Thymus sibthorpii* EO-loaded hydrogels for the in vitro cytocompatibility assessments. A cytocompatibility test of the developed hydrogels was conducted, as has been described previously [49,50], with slight modifications. The EO-loaded hydrogels were placed into 24-well tissue culture plates and sterilized using ultraviolet irradiation for 1 h before the cell culture experiments’ initiation. NIH-3T3 fibroblasts were expanded and grown in a culture medium containing high glucose (4500 mg/L) DMEM, 10% FBS, and 1% penicillin/streptomycin. Subsequently, 50,000 cells were seeded per hydrogel and were maintained at 37 °C in a humidified atmosphere containing 5% CO_2_. Fibroblasts were allowed to grow for 1, 3, and 5 days, which were the time points of the measurements. The cell metabolic activity was conducted using the alamarBlue^TM^ assay according to the manufacturer’s protocol, and the results expressed were consistent with the reduction percentage of the alamarBlue solution at each readout day (Days 1, 3, and 5). The proliferation of the NIH-3T3 fibroblasts was carried out by Quant-iT^TM^ PicoGreen^TM^ dsDNA assay in accordance with the instructions provided by the supplier. The DNA content (ng/mL) of each sample was quantified by interpolating values from a linear standard curve.

### 2.6. Statistical Analysis

In this work, all experiments were triplicated, and the data were represented as the mean ± standard deviation. One-way analysis of variance (ANOVA) was performed using GraphPad Prism^®^, Version 9.0 (La Jolla, CA, USA) after confirmation of the assumptions of the parametric analysis. Statistical significance was accepted at *p* < 0.05. The symbols * and ^#^ denote a statistically significant difference among different experimental groups and a statistically significant difference in an individual group compared to the positive control GTA, respectively. The levels of statistically significant difference were indicated as follows: * or ^#^ for *p* < 0.05, ** or ^##^ for *p* < 0.01, *** or ^###^ for *p* < 0.001, and **** or ^####^ for *p* < 0.0001.

## 3. Results

### 3.1. Determination of the Optimal starPEG Type and Concentration on Hydrogel Stability

**Figure 1:**
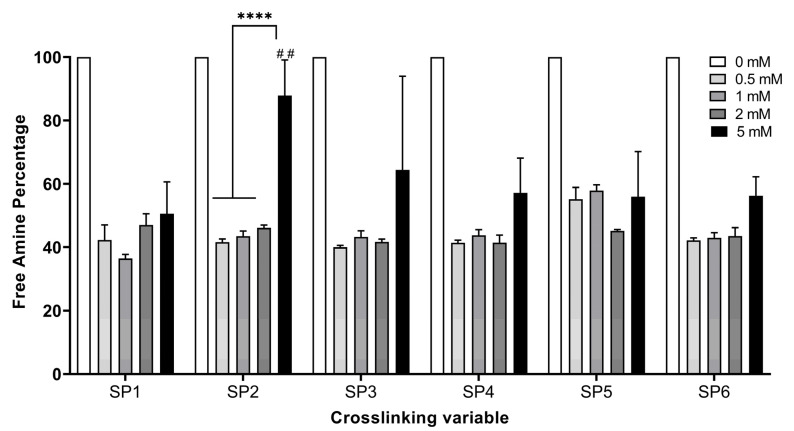
Free amine content percentage (%) of non-crosslinked (NCL) and several starPEG crosslinked collagen type I hydrogels. The concentrations of the six different PEG succinimidyl glutarate crosslinkers that were used are indicated with different colors (*n* = 3, one-way ANOVA, *p* < 0.05). The symbols * and ^#^ denote a statistically significant difference among different experimental groups and a statistically significant difference in an individual group compared to the positive control GTA, respectively. The levels of statistically significant difference were indicated as follows: ^##^ for *p* < 0.01, and **** for *p* < 0.0001.

**Figure 2:**
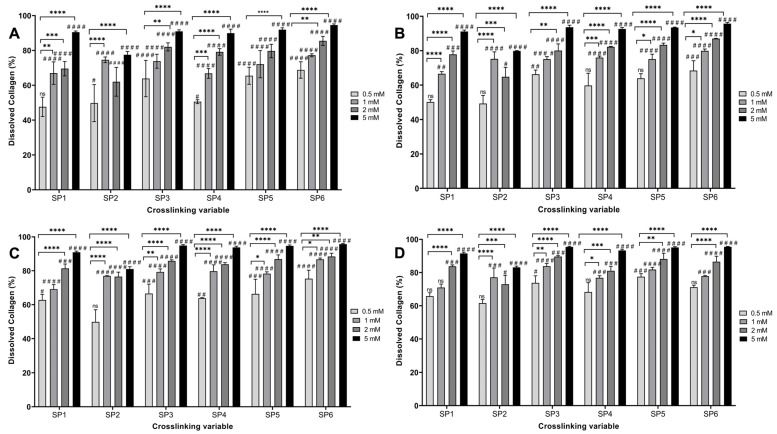
The percentage (%) of dissolved collagen of several starPEG crosslinked collagen type I hydrogels after (**A**) 2 h, (**B**) 4 h, (**C**) 8 h, and (**D**) 24 h of digestion by collagenase (n = 3, one-way ANOVA, *p* < 0.05). The concentrations of the six different PEG succinimidyl glutarate crosslinkers that were used are indicated with different colors. The symbols * and ^#^ denote a statistically significant difference among different experimental groups and a statistically significant difference in an individual group compared to the positive control GTA, respectively. The levels of statistically significant difference were indicated as follows: * or ^#^ for *p* < 0.05, ** or ^##^ for *p* < 0.01, *** or ^###^ for *p* < 0.001, and **** or ^####^ for *p* < 0.0001.

The TNBSA assay was performed to assess the free amine content of the fabricated hydrogels functionalized with various starPEG crosslinkers with 0.5, 1, 2, and 5 mM concentrations (Table 1). The starPEG-crosslinked hydrogels presented significantly decreased the free amine content for all types of crosslinkers with all tested concentrations compared to NCL hydrogels (Figure 1) (*p* < 0.05). An effective plateau was observed between 0.5 and 2 mM, and no statistical difference was noted among the 0.5 mM, 1 mM, and 2 mM crosslinked hydrogels (*p* < 0.05. In this plateau, the free amine reduction percentage was between 44.82% and 58.57%. The resistance of the hydrogels against enzymatic degradation was evaluated by the bacterial collagenase assay followed by the Pierce^TM^ BCA protein assay (Figure 2). Non-crosslinked hydrogels were completely degraded within a couple of hours. From a general perspective, scaffolds showed higher resistance to degradation when crosslinked with GTA, which was used as a positive control. However, hydrogels crosslinked with 0.5 mM of 4SP, pentaerythritol, 10 kDa showed no statistical difference compared to GTA crosslinked hydrogels, whilst 0.5 mM of 4SP, pentaerythritol, 20 kDa and 8SP, hexaglycerol, 20 kDa displayed the lowest significant differences compared to all the other groups (*p* < 0.05). Therefore, 0.5 mM of the above three crosslinkers were deemed to be optimal conditions for functionalizing collagen hydrogels.

### 3.2. EO Release Profile and Release Kinetics

**Figure 3:**
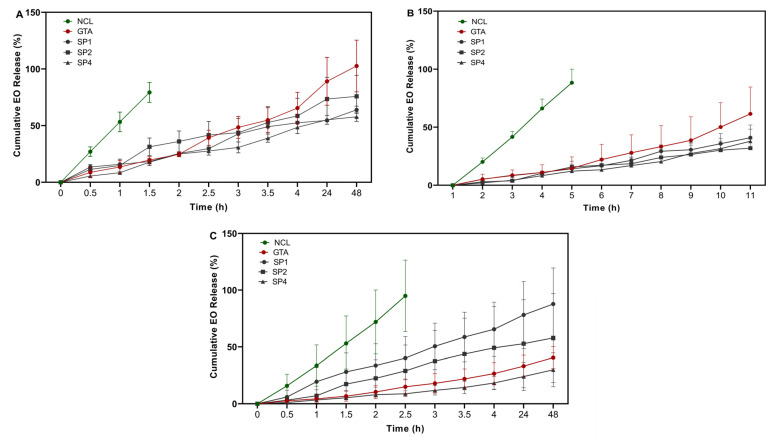
The percentage (%) of the cumulative release of *Thymus sibthorpii* essential oil loaded in optimally crosslinked collagen type I hydrogels in 1× PBS at 37 °C at concentrations of (**A**) 0.5% *v*/*v*, (**B**) 1% *v*/*v*, and (**C**) 2% *v*/*v*.

*Thymus sibthorpii* EO was loaded at 0.5, 1, and 2% *v*/*v* into optimized collagen hydrogels, and their release profile was assessed spectrophotometrically (Figure 3). The non-crosslinked hydrogels demonstrated burst release, and completely released EO within a couple of hours was observed. Although the GTA crosslinked scaffolds released almost all the loaded quantity of the EO at 0.5% *v*/*v* from the polymeric network, the chosen optimized hydrogels crosslinked with 0.5 mM of 4SP, pentaerythritol, 10 kDa, 4SP, pentaerythritol, 20 kDa, and 8SP, hexaglycerol, 20 kDa, released 63.92 ± 3.31%, 75.85 ± 9.00%, and 57.82 ± 4.08% of the EO loaded at the same concentration after 48 h. Moreover, the release kinetics were studied by applying five different mathematical models. The Hixson–Crowell model did not fit any of the experimental group, whilst the other four models fitted to different experimental groups. Moreover, the release exponent (n) values evaluated by the Korsmeyer–Peppas model indicated that the release mechanism of EO from crosslinked hydrogels obeyed the Fickian diffusion.

### 3.3. Antimicrobial Activity Analyses of the EO-Loaded Hydrogels

**Figure 4:**
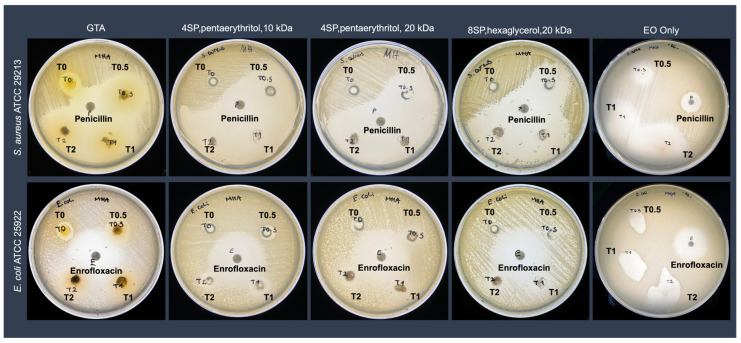
The qualitative analysis of the antibacterial effect of *Thymus sibthorpii* essential oil loaded in optimally crosslinked collagen type I hydrogels at a concentration of 0.5% *v*/*v* (T0.5), 1% *v*/*v* (T1), and 2% *v*/*v* (T2) against *S. aureus* ATCC 29213 and *E. coli* ATCC 25922. The antibiotics penicillin and enrofloxacin were used as a positive control for the *S. aureus* and *E. coli* strains, respectively. Glutaraldehyde (GTA) and antibiotics or EO-impregnated discs were used as a positive control.

**Figure 5:**
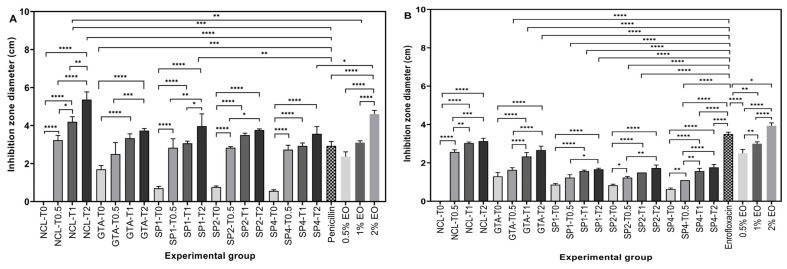
Inhibition zones’ diameters as a result of *Thymus sibthorpii* essential oil release from optimally crosslinked collagen type I hydrogels at a concentration of 0.5% *v*/*v* (T0.5), 1% *v*/*v* (T1), and 2% *v*/*v* (T2) or EO-impregnated discs against (**A**) *S. aureus* ATCC 29213 and (**B**) *E. coli* ATCC 25922 (n = 3, one-way ANOVA, *p* < 0.05). Penicillin and enrofloxacin were used as a positive control for the *S. aureus* and *E. coli* strains, respectively. Glutaraldehyde (GTA) was used as a positive control. NCL was used as a negative control. The symbol * denotes a statistically significant difference among different experimental groups and a statistically significant difference in an individual group compared to the positive control GTA. The levels of statistically significant difference were indicated as follows: * for *p* < 0.05, ** for *p* < 0.01, *** for *p* < 0.001, and **** for *p* < 0.0001.

**Figure 6:**
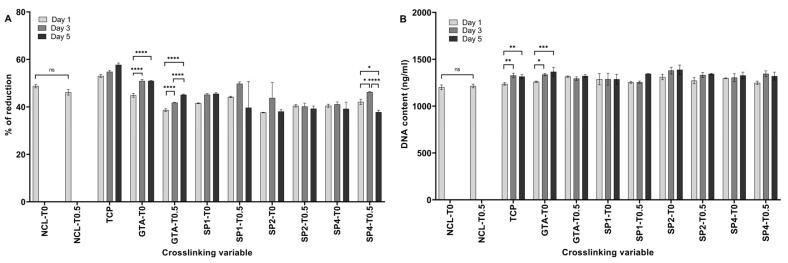
(**A**) The in vitro metabolic activity and (**B**) DNA content of the NIH-3T3 fibroblast cell line seeded on optimally crosslinked collagen type I hydrogels loaded with 0.5% *v*/*v Thymus sibthorpii* essential oil (n = 3, one-way ANOVA, *p* < 0.05). The incubation period is indicated with different colors. Glutaraldehyde (GTA) was used as a positive control. NCL was used as a negative control. TCP means a tissue culture plate where a well of a tissue culture plate contained seeded fibroblasts at the same concentration. The symbol * denotes a statistically significant difference among different experimental groups and a statistically significant difference in an individual group compared to the positive control GTA, respectively. The levels of statistically significant difference were indicated as follows: * for *p* < 0.05, ** for *p* < 0.01, *** for *p* < 0.001, and **** for *p* < 0.0001.

The antimicrobial activity of the EO-loaded hydrogels was investigated against Gram-positive *S. aureus* and Gram-negative *E. coli* using the Kirby–Bauer disc diffusion assay. The results were presented qualitatively (Figure 4) and quantitatively (Figure 5). Composite antibacterial hydrogels were less effective against Gram-negative *E. coli*. More specifically, hydrogels crosslinked with 0.5 mM 4SP, pentaerythritol, 10 kDa and loaded with 0.5 v% EO demonstrated 2.83 ± 0.47 cm and 1.23 ± 0.15 cm inhibition zone diameters against *S. aureus* and *E. coli*, respectively. Moreover, hydrogels containing 0.5% *v*/*v* of EO, which was the minor concentration, did not show a statistical difference regarding their antimicrobial activity compared to positive control penicillin (for Gram-positive bacteria) and enrofloxacin (for Gram-negative bacteria) (*p* < 0.05). Hence, 0.5% *v*/*v* concentration of *Thymus sibthorpii* EO was chosen as the optimal concentration to incorporate into collagen scaffolds. Thereafter, the cytocompatibility of 0.5% *v*/*v* EO-incorporated hydrogels was assessed on the NIH-3T3 fibroblast cell line. According to the cell metabolic activity and proliferation studies, none of the fabricated hydrogels showed any toxicity on the fibroblasts (Figure 6, *p* < 0.05).

## 4. Discussion

Antimicrobial resistance has been stated as one of the three critical public health threats by the World Health Organization (WHO) [5]. On the other hand, infections caused by antimicrobial-resistant microorganisms have been reported as the third major disease after cardiovascular diseases [51]. According to the report published by the Centers for Disease Control and Prevention (CDC), antimicrobial-resistant infections have led to the deaths of over twenty-three thousand people among the more than two million people who were infected [52]. It is expected that ten million people are going to become infected by antimicrobial-resistant microbes by 2050 according to a study published in January 2023 [53]. Therefore, the need for alternative treatments substitutes for antibiotic-based treatments to combat antimicrobial resistance has significantly gained attention. In this context, essential oils have emerged as promising alternatives with superior antimicrobial activities [54,55,56]. Although EOs have shown spectacular biological activities, they need to be incorporated in a carrier due to their high volatility and sensitivity [24]. In this study, firstly, we screened the crosslinking efficacy of six different starPEG molecules with their various concentrations to stabilize collagen type I hydrogels via in situ crosslinking. Subsequently, the optimally starPEG-crosslinked collagen hydrogels were loaded with various concentrations of *Thymus sibthorpii* EO, and the release profile and release kinetics of the EO were examined. Finally, the antimicrobial activity and cytocompatibility of the developed hydrogels were assessed.

Collagen-type I-based medical devices (e.g., hydrogels, sponges, and nanofibers) have customarily been used for tissue engineering applications thanks to outstanding properties of collagen, such as bioactivity, biocompatibility, versatility, and ability to mimic a natural extracellular matrix [25,33,57,58]. However, to enhance the stability and control the biodegradation rate and release profile of loaded drugs, collagen devices need to be introduced by crosslinking [59]. Even though enzymatic [60] and physical [61] crosslinking approaches have been studied, chemical crosslinking is generally needed for higher resistance against (bio)degradation, which creates a covalently bonded polymeric network [62,63]. Among the most studied crosslinking agents, glutaraldehyde and carbodiimide may often show some drawbacks, such as poor cell attachment, growth and proliferation, and cytotoxicity. As the literature reveals, four-arm starPEG molecules have been studied as a crosslinking agent and indicated the enhanced stability of collagen-based hydrogels [39,64]. Herein, we assessed the influence of diverse concentrations (0.5, 1, 2, and 5 mM) of the six different starPEG crosslinkers that have different arm numbers (four or eight), molecular weights (10 or 20 kDa), and functional groups (Table 1) on the stability of the collagen hydrogels. starPEG has a pegylated structure that shows multi-arm N-hydroxy succinimidyl (NHS) groups. The reactive NHS groups are expected to react with the free amine groups in the collagen backbone, consequently improving the stability of the three-dimensional collagen network. All six kinds of starPEG crosslinkers significantly decreased the free amine groups compared to non-crosslinked collagen type I hydrogels (*p* ≤ 0.05) and showed an effective plateau between 0.5 and 2 mM according to the free amine analysis (Figure 1). It is believed that 5 mM exceeds the effective concentration range for starPEG crosslinkers. In other words, the increase in the concentration to 5 mM led to higher free amine groups, which indicated a lower crosslinking efficacy. While the proven optimal concentration of GTA [40] decreased the free amine content of the collagen hydrogels by approximately 41%, 0.5, 1, and 2 mM of the studied starPEG crosslinkers showed a higher decrease with all the concentrations. In addition, 0.5 mM of all the crosslinkers except 8SP, tripentaerythritol, 10 kDa showed an approximately 60% reduction in the free amine groups. Moreover, neither molecular weight nor arm number of starPEG molecules showed a significant effect on the hydrogel stability. According to the collagenase assay, the non-crosslinked hydrogels completely degraded within 4 h due to their low stability, whilst the starPEG crosslinked hydrogels were not degraded even after 24 h (Figure 2). Similar to the free amine quantification results, a 5 mM concentration of each starPEG crosslinker showed lower efficacy than 0.5, 1, and 2 mM of the crosslinkers. The lower resistance of 5 mM starPEG crosslinked collagen hydrogels may also be explained by the self-assembly behavior of star-shaped PEG molecules. Likewise, Collin et al. revealed that the increased concentration of a four-arm starPEG crosslinker displayed a detrimental effect on the collagen type II hydrogel stability [39]. Since the 0.5 mM starPEG concentration did not show any significant difference compared to the 1 and 2 mM concentrations, it was deemed optimal. Furthermore, among the screened starPEG molecules, 4SP-pentaerythritol, 10 kDa, 4SP-pentaerythritol, 20 kDa, and 8SP-hexaglycerol, 20 kDa were chosen as the optimal crosslinkers according to the hydrogel stability outcomes.

The release profile of *Thymus sibthorpii* EO from starPEG-crosslinked hydrogels was assessed by UV–Vis spectroscopy., and 0.5, 1, and 2% *v*/*v* of the EO according to the total hydrogel volume (300 µL) was loaded into collagen hydrogels. The release behavior was examined for 0.5 h to 48 h at 37 °C. During the defined period of time, *Thymus sibthorpii* EO-loaded collagen hydrogels displayed a constant release profile (Figure 3). Since the crosslinking density and chemical composition are the key parameters for a hydrogel [65] network that can directly influence the release profile, non-crosslinked hydrogels showed burst release within a few hours, as expected. We suppose that, after crosslinking, collagen hydrogels became denser, which slowed down the EO release because of the reduced pore size and limited diffusion pathways [66]. On the other hand, the initial loading capacity of an antimicrobial agent into a polymeric network may lead to prolonged release [67]. For instance, hydrogels crosslinked with 4SP, pentaerythritol, 10 kDa approximately released 60% and 40% of the 0.5% *v*/*v* and 1% *v*/*v* loaded EO, respectively, at the end of the 12 h. In this context, it is important to understand the EO release mechanism of the hydrogel-based polymeric network. Therefore, zero-order [43], first-order [44], Higuchi [45], Korsmeyer–Peppas [46], and Hixson–Crowell [47] release kinetics models have been applied to the release data. According to the regression coefficient of the applied mathematical models (Table 2), the Hixson–Crowell model did not fit the EO-loaded various collagen systems. It was an expected outcome, since the Hixson–Crowell model mainly describes the release of a drug from systems where the change in surface area is an important parameter. On the other hand, the release exponent (n) assessed using the Korsmeyer–Peppas model is a key parameter to examine the diffusion of a drug from the polymeric networks. According to this model, for a spherical matrix, the cases n ≤ 0.45, 0.45 < n < 0.89, and n ≥ 0.89 indicate Fickian diffusion, non-Fickian diffusion, and Case-II transport, respectively. In our study, except for non-crosslinked hydrogel systems, all developed EO-loaded hydrogel systems represented Fickian diffusion, which means there are no boundaries for the release of the drug from the polymeric network. In other words, if a system obeys Fickian diffusion, a drug within the system can dissolve from any part of the polymeric matrix. Similarly, in a study, Unalan et al. loaded clove EO into the alginate/xanthan gum hydrogels and revealed that the release of the incorporated EO showed Fickian diffusion [66].

In our previous study, *Thymus sibthorpii* EO showed extraordinary antimicrobial activity against both antibiotic-resistant and non-resistant *S. aureus* strains [22]. Therefore, it was used as an antimicrobial agent in this work. On the other hand, *S. aureus* and *E. coli* are two of the mostly inhabited Gram-positive and Gram-negative bacteria on the infected tissue area [68,69,70]. Hence, the antimicrobial activity of the developed composite hydrogels was assessed against *S. aureus* and *E. coli* using the disc diffusion method. Although pristine collagen hydrogels showed antimicrobial activity, EO-loaded hydrogels presented significantly higher activity against both Gram-positive and Gram-negative bacteria (Figure 4 and Figure 5). This outcome may be explained by the antibacterial action mechanism of *Thymus sibthorpii* EO. The EO might damage the bacterial cell membrane, which becomes permeable, and the diffusion through the membrane leads to cell death [71,72]. Gram-negative bacteria have been considered more resistant to an antimicrobial agent compared to Gram-positive bacteria since their double-layered cell membrane is denser than the single-layered cell membrane of Gram-positive bacteria [73]. Accordingly, the developed antibacterial hydrogels exhibited higher antimicrobial activity against Gram-positive *S. aureus*. Additionally, some of the essential oils could diffuse through the lipophilic cell wall of Gram-negative bacteria (e.g., *E. coli*) due to the diverse chemical composition of essential oils, which could explain the observed antimicrobial action of composite collagen hydrogels. For instance, Aras et al. [74] developed *Nigella sativa* EO-incorporated polyurethane-based nanofibrous mats. The developed wound dressings showed higher antibacterial activity on *E.coli* than on *S. aureus* [74]. We used penicillin and enrofloxacin as control antimicrobial agents for the comparison of the efficacy of *Thymus sibthorpii* EO on *S. aureus* and *E. coli*, respectively. The hydrogels crosslinked with all three different starPEG and loaded with 0.5% *v*/*v* EO did not show any significant difference compared to the control antimicrobials. For this reason, the 0.5% *v*/*v Thymus sibthorpii* EO concentration was deemed optimal to incorporate into collagen type I antibacterial hydrogels. We believe that, since even the lowest concentration of *Thymus sibthorpii* EO can be used instead of penicillin, *Thymus sibthorpii* EO can be presented as an alternative and effective antimicrobial agent to overcome the antimicrobial resistance problem raised by the misuse and long-term use of antibiotics.

Collagen-based medical devices are widely used for tissue engineering applications, because they show advanced biocompatibility with mammalian cells in addition to other outstanding features [58,75]. Among the collagen-based scaffolds, collagen hydrogels demonstrate cell attachment, proliferation, and metabolic activity due to their porous and fibrillar network [76,77]. The optimized 0.5% *v*/*v Thymus sibthorpii* EO-loaded collagen hydrogels crosslinked with 4SP-pentaerythritol, 10 kDa, 4SP-pentaerythritol, 20 kDa, and 8SP-hexaglycerol, 20 kDa were examined by the alamarBlue^TM^ and Quant-iT^TM^ PicoGreen^TM^ dsDNA assays for metabolic activity and the proliferation of the seeded NIH-3T3 fibroblasts, respectively. The experimental outcomes indicate that all of the developed composite hydrogels were found cytocompatible with fibroblasts. Moreover, it can be concluded that a 0.5 v% concentration of *Thymus sibthorpii* EO has no toxic effect on the NIH-3T3 fibroblasts.

## 5. Conclusions

Antimicrobial resistance is an emerging global health threat that causes drastically increasing mortality and economic burden every year. Consequently, alternative safer antibacterial therapy strategies need to be taken into consideration to combat this threat. In the quest for alternative antibacterial therapies, herein, we developed collagen type I hydrogel systems optimally crosslinked and loaded with *Thymus sibthorpii* essential oil. The proposed antimicrobial agent incorporated into the collagen type I scaffolds showed strong activity against *S. aureus*, demonstrated a sustained release profile, and had no toxicity on fibroblasts. The outcomes of this work make this developed composite antibacterial medical device a promising candidate for infected tissue engineering applications. This study will be continued by conducting detailed material characterization (e.g., porosity measurement, FTIR analysis, mechanical testing, DSC analysis, and SEM imaging) and investigating any influence of essential oil on the material properties.

## Figures and Tables

**Table 1:** The arm number, functional group, molecular weight (MW), and used concentrations of six different PEG succinimidyl glutarate crosslinkers that were used for the stabilization of collagen type I hydrogels in the study.

Full Name	Abbreviation	Code	Arm Number	Functional Group	MW (kDa)	Concentration (mM)
4-arm PEG Succinimidyl Glutarate, pentaerythritol, 10 kDa	4SP, pentaerythritol, 10 kDa	SP1	4	pentaerythritol	10	0, 0.5, 1, 2, 5
4-arm PEG Succinimidyl Glutarate, pentaerythritol, 20 kDa	4SP, pentaerythritol, 20 kDa	SP2	4	pentaerythritol	20	0, 0.5, 1, 2, 5
8-arm PEG Succinimidyl Glutarate, hexaglycerol, 10 kDa	8SP, hexaglycerol, 10 kDa	SP3	8	hexaglycerol	10	0, 0.5, 1, 2, 5
8-arm PEG Succinimidyl Glutarate, hexaglycerol, 20 kDa	8SP, hexaglycerol, 20 kDa	SP4	8	hexaglycerol	20	0, 0.5, 1, 2, 5
8-arm PEG Succinimidyl Glutarate, tripentaerythritol, 10 kDa	8SP, tripentaerythritol, 10 kDa	SP5	8	tripentaerythritol	10	0, 0.5, 1, 2, 5
8-arm PEG Succinimidyl Glutarate, tripentaerythritol, 20 kDa	8SP, tripentaerythritol, 20 kDa	SP6	8	tripentaerythritol	20	0, 0.5, 1, 2, 5

**Table 2:** Regression coefficients (R^2^) of the five different release kinetic models fitted to the release of three different concentrations of *Thymus sibthorpii* EO from each starPEG-crosslinked collagen type I hydrogels and the controls. T0.5, T1, and T2 represent the 0.5, 1, and 2% *v*/*v* concentrations of *Thymus sibthorpii* essential oil loaded in hydrogels.

Model	Zero-Order	First-Order	Higuchi	Korsmeyer–Peppas		Hixson–Crowell
	R^2^	R^2^	R^2^	R^2^	n	R^2^
NCL-T0.5	0.9996	0.9538	0.9294	1.0000	0.9789	0.4040
NCL-T1	0.9991	0.9050	0.8987	0.9996	1.0720	0.5536
NCL-T2	0.9972	0.8101	0.8821	0.9997	1.1065	0.6183
GTA-T0.5	0.9574	1.0000	0.9971	0.9985	0.1789	0.0235
GTA-T1	0.9973	0.9998	0.9897	0.9666	0.1779	0.7033
GTA-T2	0.9998	0.9997	0.9806	0.9494	0.1613	0.9434
SP1-T0.5	0.9153	0.9039	0.8113	0.6980	0.0677	0.6412
SP1-T1	0.9973	0.9991	0.9897	0.9573	0.1111	0.7033
SP1-T2	0.9829	0.9997	0.9995	0.9830	0.1140	0.1856
SP2-T0.5	0.8124	0.8400	0.9196	0.9747	0.1082	0.0953
SP2-T1	0.9336	0.9382	0.9887	0.9996	0.0747	0.1965
SP2-T2	0.9981	0.9952	0.9615	0.8993	0.0607	0.0152
SP4-T0.5	0.9155	0.9277	0.9802	0.9999	0.0706	0.9991
SP4-T1	0.9949	0.9916	0.9502	0.8958	0.1197	0.0280
SP4-T2	0.9982	0.9996	0.9876	0.9645	0.1882	0.7830

## Data Availability

Data available in a publicly accessible repository.

## Supplementary Materials

The following supporting information can be downloaded at: https://www.mdpi.com/article/10.3390/bioengineering12010089/s1, Figure S1: Calibration curve for the TNBSA assay with the known concentrations of glycine; Figure S2: Calibration curve for the release profile assessment with the known concentrations of *Thymus sibthorpii* essential oil.
