# Supplementary material for: Release Profile and Antibacterial Activity of Thymus sibthorpii Essential Oil-Incorporated, Optimally Stabilized Type I Collagen Hydrogels
Source: Bioengineering (Basel). 2025 Jan 19;12(1):89. doi: 10.3390/bioengineering12010089 (PMC11760836; doi:10.3390/bioengineering12010089)
Supplement: Supplementary file 1 [file bioengineering-12-00089-s001.zip › bioengineering-3385261-supplementary.pdf]

Supplementary Information

Release Profile and Antibacterial Activity of *Thymus sibthorpii* Essential Oil-Incorporated, Optimally Stabilized Type I Collagen Hydrogels

Caglar Ersanli (1,2,3), Ioannis Skoufos (1), Konstantina Fotou (2), Athina Tzora (2), Yves Bayon (4), Despoina Mari(5,6), Eleftheria Sarafi(5,6), Konstantina Nikolaou (2) and Dimitrios I. Zeugolis (3)

1) Laboratory of Animal Science, Nutrition and Biotechnology, School of Agriculture, University of Ioannina, 47100 Arta, Greece.
2) Laboratory of Animal Health, Food Hygiene and Quality, School of Agriculture, University of Ioannina, 47100 Arta, Greece.
3) Regenerative, Modular & Developmental Engineering Laboratory (REMODEL), Charles Institute of Dermatology, Conway Institute of Biomolecular and Biomedical Research and School of Mechanical and Materials Engineering, University College Dublin (UCD), Dublin, Ireland.
4) Medtronic, Trevoux, France.
5) Department of Biological Applications & Technology, School of Health Sciences, University of Ioannina, 45110 Ioannina, Greece.
6) Biomedical Research Institute, Foundation for Research and Technology-Hellas, 45110 Ioannina, Greece.

Information about our type I collagen

Type I porcine collagen is extracted from pig dermis. The telopeptides are removed by pepsin, which also helps to eliminate proteins and/or polypeptides as contaminants. The molar mass of the obtained collagen is approximately 100 kDa, with the expected ratio of 2 to 1 for the alpha-1 ($\alpha 1$) and alpha-2 ($\alpha 2$) chains. The collagen is thermally non-denatured, exhibiting a Differential Scanning Calorimetry (DSC) profile typical of type I collagen and showing no visible hydrolysis bands by electrophoresis. The purity is estimated to be over 95% based on electrophoresis analysis, which visibly shows only the expected bands of type I collagen. The solubility and filterability rates of the collagen are above 95%, and the hydroxyproline content is above 11% by mass.

Calibration curves

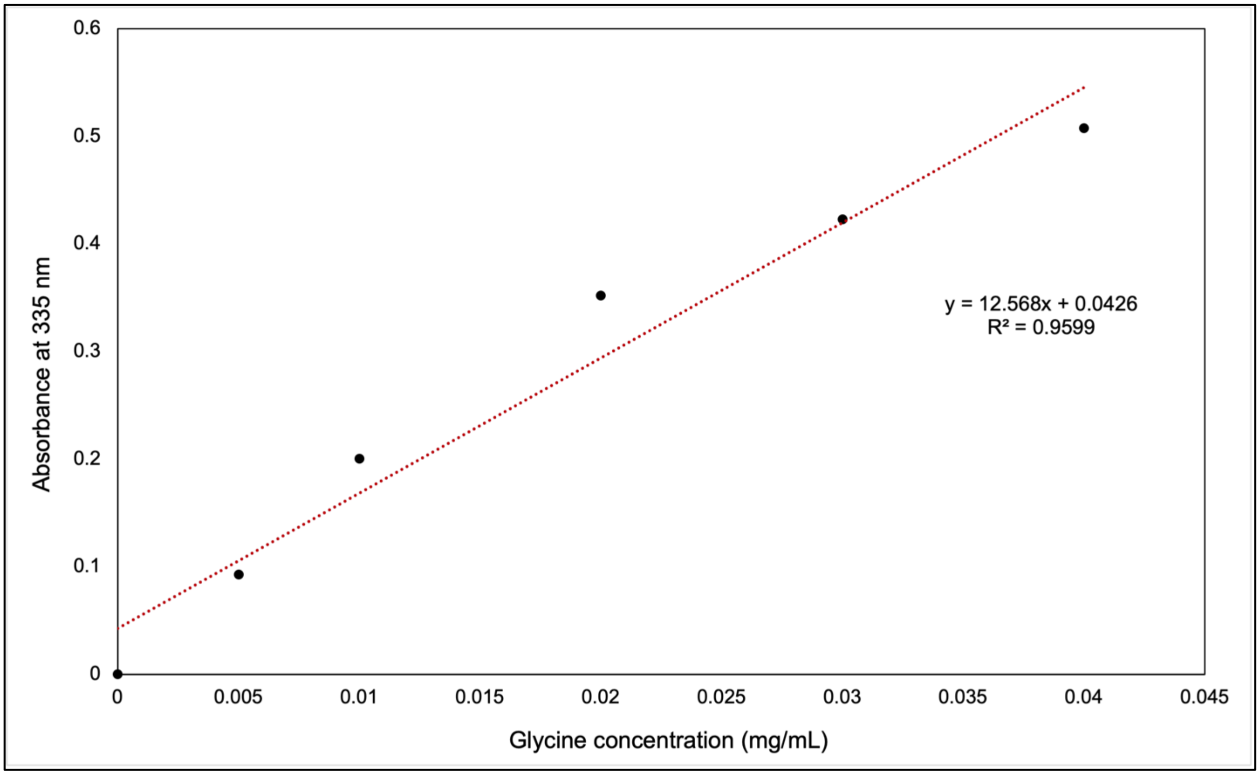

Supplementary Figure S1. Calibration curve for the TNBSA assay with the known concentrations of glycine.

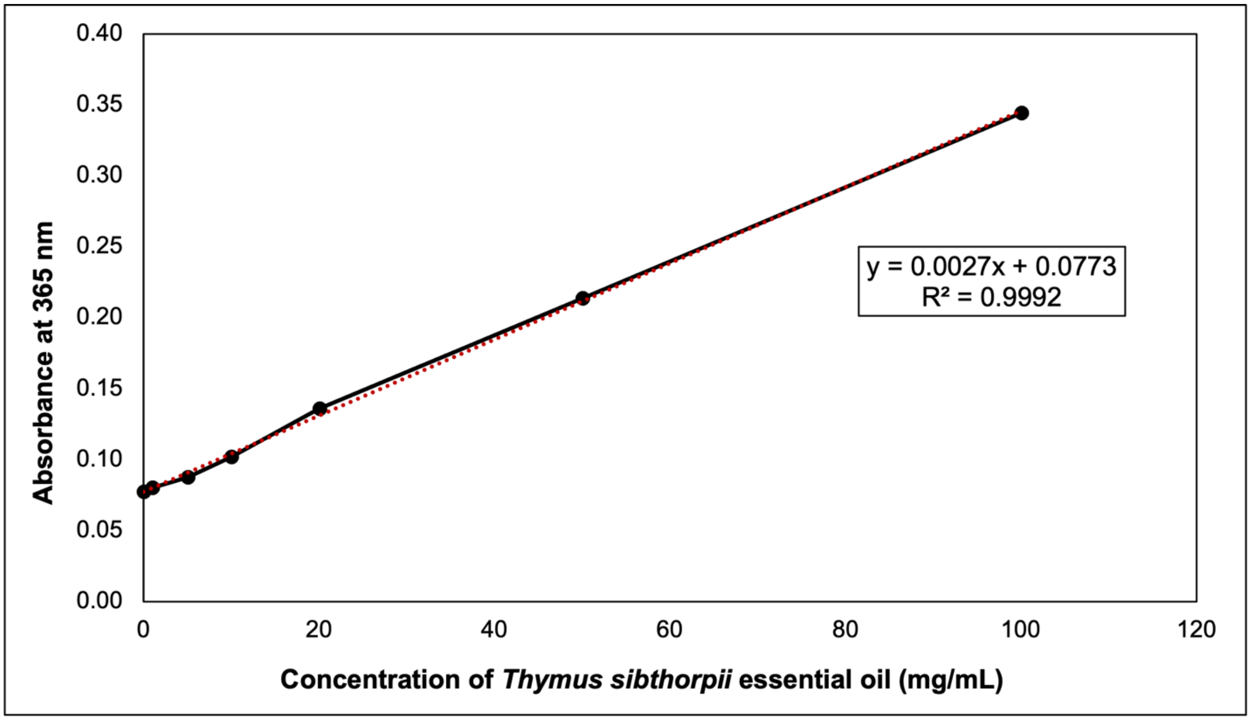

Supplementary Figure S2. Calibration curve for the release profile assessment with the known concentrations of *Thymus sibthorpii* essential oil.
